# Mesangial Cell–Derived Exosomal miR-4455 Induces Podocyte Injury in IgA Nephropathy by Targeting ULK2

**DOI:** 10.1155/2022/1740770

**Published:** 2022-11-04

**Authors:** Mengjie Yu, Xiaogang Shen, Wenfang He, Danna Zheng, Qiang He, Juan Jin

**Affiliations:** ^1^Bengbu Medical College, Bengbu, Anhui 233000, China; ^2^Urology & Nephrology Center, Department of Nephrology, Zhejiang Provincial People's Hospital (Affiliated People's Hospital, Hangzhou Medical College), Hangzhou, Zhejiang 310014, China; ^3^Department of Nephrology, The First Affiliated Hospital of Zhejiang Chinese Medical University (Zhejiang Provincial Hospital of Traditional Chinese Medicine), Hangzhou, Zhejiang 310000, China

## Abstract

Growing evidence suggests that mesangial cells (MCs) play a crucial role in the pathogenesis of IgA nephropathy (IgAN) by secreting aIgA1. However, the mechanism by which MCs regulate podocyte injury remains unknown. This study demonstrated that MC-derived exosomes treated with aIgA1 induced podocyte injury in IgA nephropathy. miR-4455, which was significantly upregulated in aIgA1 treatment MC-derived exosomes, can be transferred from MCs to podocytes via exosomes. MC-derived exosomal miR-4455 induced podocyte injury. Mechanistically, exosomal miR-4455 directly targeted ULK2 to regulate LC3II/I and P62 levels, which mediates autophagy homeostasis. This study revealed that MC-derived exosomal miR-4455 is a key factor affecting podocyte injury and provides a series of potential therapeutic targets for treating IgA nephropathy.

## 1. Introduction

IgAN is the most common primary glomerulopathy worldwide, causing lifelong disease [1]. Up to 30 percent of cases progress toward end-stage renal disease (ESRD), requiring renal replacement therapy [2]. An important element in IgAN pathogenesis is the formation of IgA1 immune complexes [3] because the primary site of IgA1 immune complex deposition is the mesangium; mesangial cell (MC) proliferation and matrix excess deposition are the first histopathologic lesions [4] observed in disease development. Moreover, future deposition of these aberrant IgA1 immune complexes in the mesangial area induces localized damage and progressive renal dysfunction [5]. Podocytopathic changes are a consequence of initial alterations in the mesangial area with the accumulation of IgA1 containing immune complexes. It has been shown that MCs and podocytes are the main cell types damaged by IgAN. Podocyte injury has become increasingly accepted as a key mechanism leading to disease progression in IgAN [6–8], and the severity of IgAN disease increases as the number of podocytes decreases.

In recent years, the role of exosomes in IgAN has become an important research topic [9, 10]. Exosomes are cell-secreted extracellular vesicles with diameters of 30–150 nm that have been reported as important regulators of intercellular signaling [11] and promising biomarkers [12]. Exosomes transport complex biological molecules including proteins, lipids, DNA, and RNA [13]. Among these cargo types, microRNAs (miRNAs) are among the most important signaling molecules: (1) miRNAs are 21–25 nucleotides in length and can be efficiently packaged into exosomes, (2) miRNAs are stably distributed in various tissues and body fluids, and (3) a single miRNA can regulate multiple target genes. Conversely, multiple miRNAs may act synergistically to regulate a single gene, leading to an efficient and flexible gene regulation pattern [14]. We [15] and other groups [16] have confirmed that exosome-mediated delivery of functional miRNAs plays a key role in intercellular communication in podocyte injury.

In this study, exosomes were successfully isolated from MCs treated with aIgA1, and MC-derived exosomal miR-4455 was found to induce podocyte injury. Mechanistically, MC-derived exosomal miR-4455 directly targets ULK2 and regulates LC3II/I and P62 levels, thereby weakening podocyte autophagy and aggravating podocyte injury. This suggests that miR-4455 plays a key role in inducing podocyte injury and elucidates a potential therapeutic target for IgA nephropathy.

## 2. Methods and Materials

### 2.1. Cell Culture

Human primary podocytes were purchased from OTWO, China, HTX2426 and cultured in RPMI1640 (Procell, China, PM150110) containing 20 U/mL *γ*-IFN, 10% fetal bovine serum (Procell, China, 164210-500), and 1% P/S (Procell, China, PB180120) at 33°C and 5% CO_2_. Then, cells were cultured at 37°C for 10 days to induce podocyte maturation. Human glomerular MCs (HMC cells) were purchased from Procell (China, CL-0619) and cultured in Dulbecco's Modified Eagle Medium (Procell, China, PM150210) containing 10% FBS (Procell, China, 164210-500) and 1% P/S (Procell, China, PB180120) at 37°C and 5% CO_2_.

### 2.2. Exosome Extraction

Human-derived IgA1 protein (Abcam, ab91020) was heated and treated on a drying plate at 63°C for 150 min, yielding aIgA1 [17]. HMC cells were treated with solvent or 25 *μ*g/mL aIgA1 for 24 h, fresh medium without solvent and aIgA1 was replaced, and the supernatant was collected after 24 h of culture. Exosomes were extracted using a Japan Heguang (293-77601) exosome extraction kit.

### 2.3. Electron Microscopic Observation of Exosomes

After extraction, 10 *μ*l of purified exosomes was isolated and diluted by adding an equal volume of balanced salt PBS solution and then added dropwise onto a 2 mm carrier copper grid. The grid was left at room temperature (25°C) for 1 min, and the excess liquid was gently aspirated with filter paper, negatively stained with 3% (w/v) sodium phosphotungstate solution (pH 6.8) at room temperature for 5 min, washed with double distilled water, dried at room temperature, observed using a transmission electron microscope (JEOL LTD, JEM-1400Plus), and photographed.

### 2.4. Nanoparticle Tracking Analysis

The exosomes were diluted (1 : 10) in PBS for nanoparticle tracking analysis (NTA) using a nanoparticle tracking analyzer (Particle Metrix, ZetaView). The Brownian motion of each particle was tracked between frames, and the size was calculated by the Stokes-Einstein equation.

### 2.5. Detection of Exosome Uptake by Podocytes

PKH26 kit (Sigma, MINI26-1KT) was used to detect the exosome uptake ability of podocytes. 1 × 10^5^ cells/well were collected in the logarithmic growth phase, in good condition, and plated on a 12-well plate. Exosomes were cultured at 37°C overnight and then centrifuged at 12000 rpm for 10 min to remove the supernatant. 0.5 mL of diluent C was added to prepare a 2× exosome suspension. A 2× dyeing solution was prepared by adding 2 *μ*L PKH26 ethanol solution into another 0.5 mL diluent C. The 2× exosome suspension was added to the 2× staining solution, the solution was mixed, and the stained cells were incubated for 5 min. An equal volume of serum was added to terminate the reaction and incubated for 1 min to bind the excess dye. The solution was centrifuged at 12,000 rpm for 10 min, and the supernatant was removed. Exosomes were cleaned twice with complete medium to remove unbound dyes and washed 3 times with PBS, for 3 min. DAPI staining solution was added to the slide, incubated at room temperature (25°C) for 5 min, and then washed with PBS 3 times, for 3 min each time. Slides were sealed with mounting solution containing antifluorescence quencher and observed under a fluorescence microscope.

### 2.6. Quantitative Real-Time PCR

Cell culture plates were lysed for 10 min with 1 mL of TRIzol Reagent (Ambion, 15596-026). RNA was extracted using the RNeasy Mini Kit (Qiagen no. 74106). cDNA synthesis was performed using a high-volume cDNA reverse transcription kit (Applied Biosystems no. 4368813). qPCR experiments were performed using the StepOnePlus Real-Time PCR system in SYBR Green assay (Applied Biosystems), and the relative gene expression in each sample was determined using comparative CT value (*ΔΔ*CT) analysis and normalization with U6. The PCR primer sequences and related primer sequences are listed in Table 1.

### 2.7. CCK-8

Human podocytes were treated with different concentrations of MC exosomes (12.5, 25, and 50 *μ*g/mL) for 24 h. Cell viability of each treatment group was measured using a commercially available CCK-8 assay kit (Seven Seas Bio, 20150520). The absorbance of each well was measured at 450 nm using a Multiskan MK3 enzyme marker (MD, SpectraMaX M3).

### 2.8. Flow Cytometry

A flow cytometry apoptosis kit (KGA101) was used according to the following protocol: the cultured cells were digested with trypsin, washed with cold PBS, added with dyes, mixed well, and allowed to react overnight in the dark at 4°C. PBS (1 mL) was added to each tube and centrifuged at 2000 rpm for 10 min to remove unlabeled antibodies, the supernatant was discarded, and PBS (0.5 mL) was added to each tube to resuspend the cells. Apoptosis was detected using flow cytometry (Accuri C6).

### 2.9. Western Blotting

The exosomes were washed with PBS, and lysate containing phenylmethylsulfonyl fluoride (Nanjing Vohong, 329-98-6) was added (Biyuntian, P0013B) and lysed for 30 min. The lysate was transferred to a 1.5 mL centrifuge tube, and the supernatant was removed by centrifugation at 12000 rpm for 15 min at 4°C. The protein lysate concentration was measured using a Bio-Rad Protein Assay Kit II (Bio-Rad, 5000002). After, the protein lysate was mixed with 5× sample buffer (15 g SDS, 15.6 mL 2 M Tris pH 6.8, 57.5 g glycerol, and 16.6 mL b-mercaptoethanol), and the samples were placed on 10% polyacrylamide gels, separated by SDS-PAGE, and transferred to PVDF membranes (Bio-Rad no. 162-0177). After blocking with 4% milk containing 0.1% Tween, anti-CD9 antibody (1 : 2000; Abcam, ab92726), anti-CD63 antibody (1 : 1000; Abcam, ab217345), anti-CD81 antibody (1 : 1000; Abcam, ab109201), anti-ULK2 antibody (1 : 2000; Abcam, ab97695), anti-LC3 antibody (1 : 500; Proteintech, 12135-1-AP), anti-p62 antibody (1 : 500; Proteintech, 18420-1-AP), and anti-GAPDH antibody (1 : 2500; Abcam, ab9485) were incubated overnight at 4°C. The membranes were washed three times with PBS containing 0.1% Tween and then incubated with HRP-labeled secondary antibody (Dianova, Hamburg, Germany) prepared with 4% skimmed milk containing 0.1% Tween for 2 h at room temperature, washed three times with PBS containing 0.1% Tween, and then removed (Rad no. 170-5060) and placed into the GelDoc imaging system (Bio-Rad) for photography. Protein expression levels were normalized to that of the internal reference protein, GAPDH.

### 2.10. Construction of ULK2 mRNA UTR^WT^ and ULK2 mRNA UTR^MUT^ Luciferase Vectors

The pUC57-Homo-ULK2-3′UTR and pYr-MirTarget were double digested with XhoI and NotI at 37°C for 6 h, and Homo-ULK2-3′UTR and pYr-MirTarget were recovered and purified on 1% agarose gel. The purified target fragment Homo-ULK2-3′UTR (XhoI/NotI) was ligated to the purified vector pYr-MirTarget (XhoI/NotI) overnight at 4°C, and the ligated product was named pYr-MirTarget-Homo-ULK2-3′UTR. The ligated product was transformed into DH5*α* receptor cells, coated onto lysogeny broth with ampicillin (LB) plates, and incubated overnight at 37°C. Several monoclonal colonies were selected from each culture dish, inoculated in LB culture medium containing ampicillin resistance, and incubated overnight in a shaker at 37°C. The corresponding bacterial broth was used for colony PCR with the following primers: pYr-MirTarget F: GGTTCTTTTCCAACGCTATT; smad1-3′UTR R: GACTCATTTAGATCCTCAC. Mutation primer design was as follows (Homo-ULK2-3′UTR-mut-F: TAAAAATTACAAGAGCGAATTTTGATAACTTTAGTT; Homo-ULK2-3′UTR-mut-R: TTATCAAAATTCGCTCTTGTAATTTAACTTGATA). The correct bacterial broth samples were sequenced prior to plasmid extraction (pYr-MirTarget-Homo-ULK2-3′UTR and pYr-MirTarget-Homo-ULK2-3′UTR-mut).

### 2.11. Dual Luciferase Reporter Gene Assay

HEK293T cells were inoculated in a 24-well plate, transfected with plasmid for 48 h, and washed twice with PBS. Then, 200 *μ*L of reporter gene cell lysate was added to each well. Cells were collected and centrifuged at 10000–15000 g for 5 min, and the supernatant was collected. For chemiluminescence analysis, the measurement interval was set at 2 s, and the measurement time was 10 s. For measurement, 50 *μ*L of the sample was taken, 100 *μ*L of sea renin fluorophore enzyme detection reagent was added, and the relative light unit (RLU) was measured after homogenization with a gun (Eppendorf, Germany). After completing the above steps, 100 *μ*L of fluorophore enzyme assay solution was added, and the RLU was measured by gun beating.

### 2.12. ULK2 Overexpression Vector Construction

Primers were designed using NM_014683.4, and human ULK2 (NM_014683.4) cDNA was cloned into the RCR plasmid to obtain the target fragment. The plasmid pcDNA3.1 (+) and the gel recovery purification product Homo-ULK2 were double digested with BamHI and EcoRI. The double-digested vector and fragment were recovered and purified on an agarose gel, and the recovered purified target fragment was ligated to the recovered purified vector. The ligation products were transformed into DH5*α* receptor cells, coated with LB AMP plates, and incubated overnight at 37°C in a warm oven. Several monoclonal colonies were selected from the culture dish, inoculated in LB culture medium containing ampicillin resistance, and incubated overnight at 37°C in a constant temperature shaker. The corresponding bacterial solution was used for colony PCR, and the identified bacterial solution was sent for sequencing.

### 2.13. Statistical Analysis

Statistical software (version 9.0) was used for data analysis, and all data were expressed as mean ± standard deviation (^−^*X* ± *S*). The least significant difference method in one-way ANOVA was used for two-way comparisons between groups. *p* < 0.05 was considered statistically significant.

## 3. Results

### 3.1. aIgA1 Treatment Does Not Affect MC Exosome Secretion

**Figure 1:**
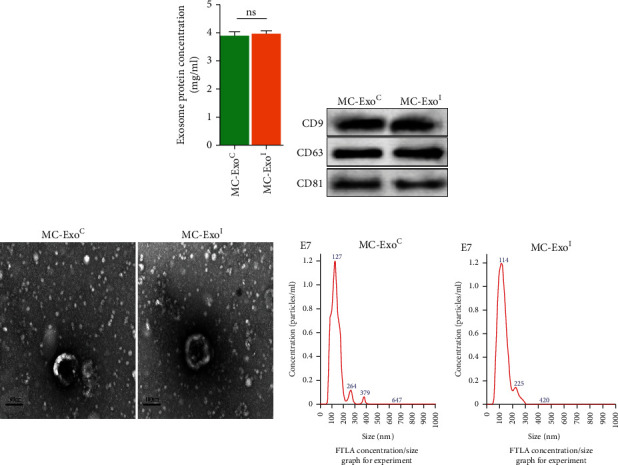
Effect of aIgA1 treatment on exosome secretion from MCs and exosome identification. (a) Concentration of exosomal protein secreted by MCs after 24 h treatment with solvent or aIgA1 were measured by BCA protein concentration assay kit. (b) Expression of CD9, CD63, and CD81 in the extracted mesangial cell exosomes was measured by western blotting. (c) Morphology and size of MC exosomes in each group were observed under a transmission electron microscope and NTA (ns means *p* > 0.05).

Exosomes (MC-ExoC or MC-ExoI) extracted from human MC treated with solvent or aIgA1 were detected by Western blotting, observed using transmission electron microscopy and NTA. The results showed that aIgA1 treatment did not change the concentration of exosome proteins in MCs (Figure 1(a)), and exosome markers CD9, CD63, and CD81 were expressed in both groups (Figure 1(b)). Transmission electron microscopy revealed that the morphology of exosomes in both groups was spherical, and NTA showed that the particle size of exosomes in MC-ExoC and MC-ExoI was 127 nm and 114 nm (Figure 1(c)), respectively, which was consistent with the particle size of exosomes (30-150 nm) [18], indicating that exosomes were successfully extracted from MCs and that aIgA1 treatment did not affect exosome secretion.

### 3.2. Exosomes Secreted by MCs Treated with aIgA1 Can Induce Podocyte Injury

**Figure 2:**
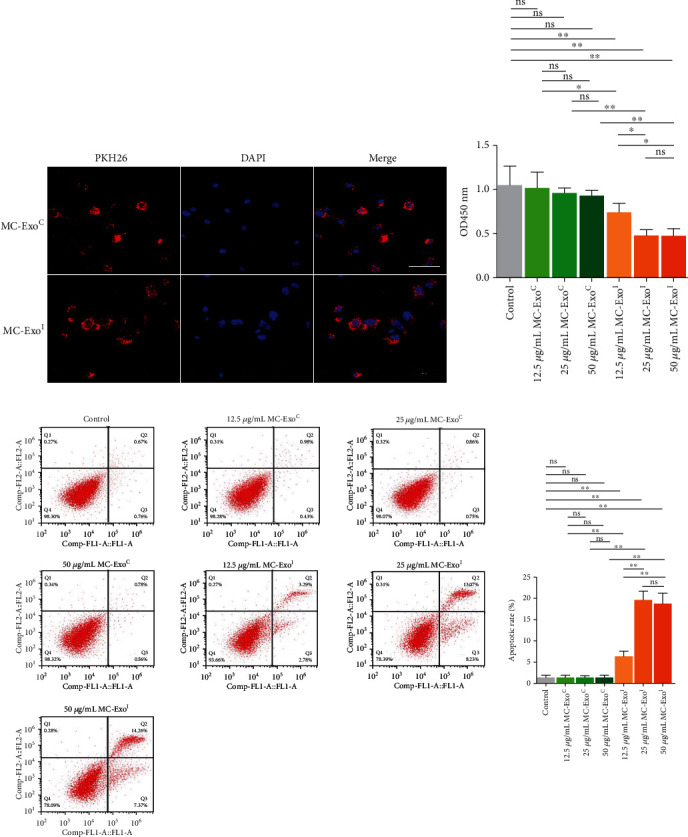
Effect of MC-ExoI on podocytes activity and apoptosis. (a) Detection of podocyte uptake capacity of two groups of exosomes. Red indicates PKH26-labeled exosomes, scale bar = 50 *μ*m. (b) Effect of MC-ExoI on podocytes activity was detected by CCK8. (c) Effect of MC-ExoI on podocytes apoptosis was detected by flow cytometry (^∗^*p* < 0.05, ^∗∗^*p* < 0.01, ns means *p* > 0.05).

To clarify the uptake ability of podocytes to MC-ExoC and MC-ExoI, podocytes were incubated with PKH26-labeled MC-ExoC and MC-ExoI, respectively. The results showed that exosomes were expressed in both groups of podocytes, and the expression intensity was consistent (Figure 2(a)). This indicates that podocytes could uptake mesangial cell-derived exosomes, and the uptake ability of MC-ExoC and MC-ExoI was consistent. To clarify the effect of exosomes secreted by MC after aIgA1 treatment on human podocyte injury, the effect was assessed by CCK-8 and flow cytometry analyses. The results showed that exosomes secreted by MCs treated with solvent (MC-ExoC) did not affect podocyte activity and apoptosis level (12.5–50 *μ*g/mL). Exosomes secreted by MCs treated with aIgA1 (MC-ExoI) significantly reduced podocyte activity and induced podocyte apoptosis. The inhibitory effect of MC-ExoI (25-50 *μ*g/mL) on podocyte activity and apoptosis was stronger than that of MC-ExoI (12.5 *μ*g/mL), but there was no significant difference between the medium- and high-concentration groups (*p* > 0.05, Figures 2(b) and 2(c)). Therefore, 25 *μ*g/mL was selected to explore the mechanism underlying exosome-induced podocyte injury.

### 3.3. Exosomes Secreted by MCs Treated with aIgA1 Induce Podocyte Injury by Upregulating miR-4455 Expression in Podocytes

**Figure 3:**
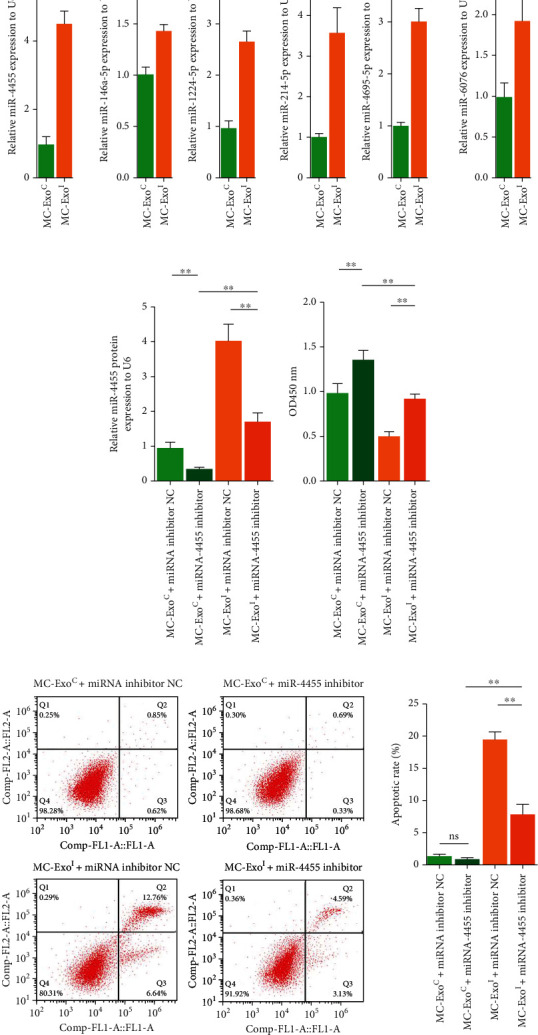
Effect of MC-ExoI and miR-4455 inhibitor on podocyte activity and apoptosis. (a) Contents of miR-4455, miR-146a-5p, miR-1224-5p, miR-214-5p, miR-4695-5p, and miR-6076 in MC-ExoC or MC-ExoI were detected by qRT-PCR. (b) MC-ExoC or MC-ExoI effect combined with miR-4455 inhibitor on miR-4455 content in human podocytes was detected by qRT-PCR. (c) MC-ExoC or MC-ExoI effect combined with miR-4455 inhibitor on human podocyte activity was detected by CCK8. (d) MC-ExoC or MC-ExoI effect combined with miR-4455 inhibitor on human podocyte apoptosis was detected by flow cytometry (^∗∗^*p* < 0.01).

Based on the literature [19], we screened the exosomes secreted by MCs, treated with secretory IgA from salivary glands of patients with IgAN, which expressed significantly upregulated TOP15 miRNAs. By analyzing the related reports of these miRNAs and cell growth and apoptosis (Table 2), six miRNAs that can promote cell injury or inhibit cell growth were screened for, including miR-4455, miR-146a-5p, miR-1224-5p, miR-214-5p, miR-4695-5p, and miR-6076. The miRNA content in MC-ExoC and MC-ExoI was detected by qRT-PCR. The expression of six miRNAs in MC-ExoI were increased compared with MC-ExoC, and the upregulation of miR-4455 content was the largest (Figure 3(a)).

To analyze the effect of MC exosomes on miR-4455 content in podocytes, podocytes were treated with an miR-4455 inhibitor. We found that the content of miR-4455 in podocytes in the MC-ExoI+miRNA inhibitor NC group was significantly upregulated, and the miR-4455 inhibitor significantly inhibited the promotion of MC-ExoI on the content of miR-4455 in podocytes (Figure 3(b)). CCK-8 and flow cytometry showed that miR-4455 inhibitor treatment significantly increased podocyte activity and inhibited podocyte apoptosis under the treatment of MC exosomes and blocked the decrease in podocyte activity and increase in podocyte apoptosis caused by MC-ExoI treatment (Figures 3(c) and 3(d)). These results indicated that MC-ExoI induced podocyte injury by upregulating the miR-4455 expression in podocytes.

### 3.4. Exosomal miR-4455 Directly Targets ULK2 to Mediate Podocyte Injury

**Figure 4:**
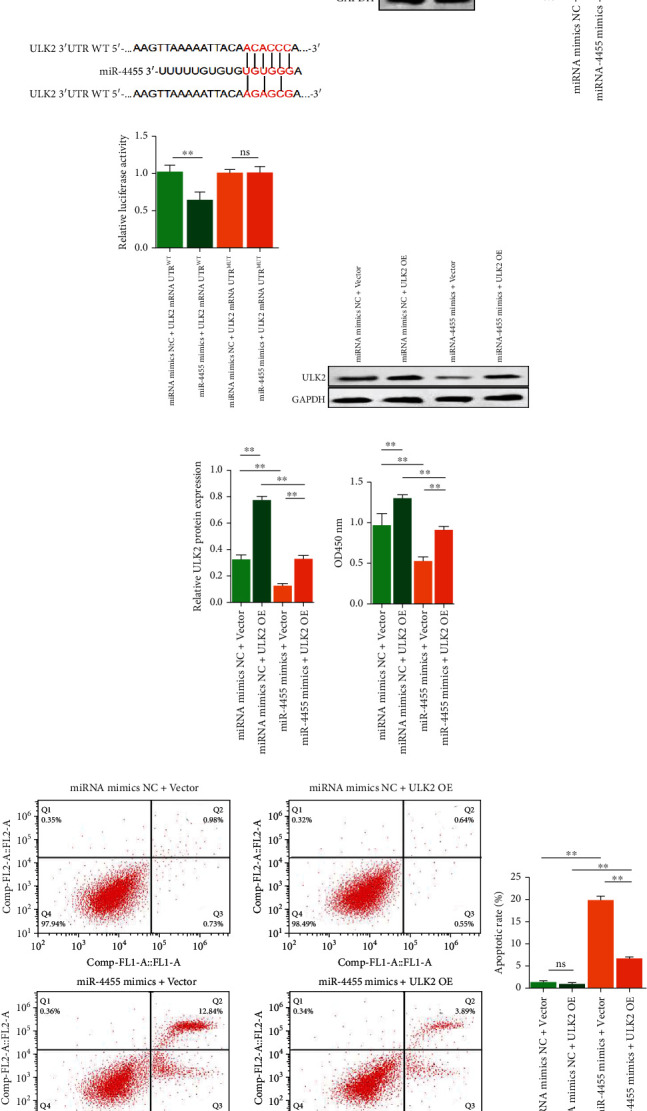
Effects of miR-4455 and ULK2 mRNA 3′UTR binding and miR-4455 mimics combined with ULK2 overexpression plasmid transfection on podocytes activity and apoptosis. (a) Dual luciferase reporter gene assay for miR-4455 and ULK2 mRNA 3′UTR binding. (b) Effect of miR-4455 mimics on the ULK2 expression in human podocytes revealed by western blotting. (c) Detection of luciferase activity of miR-4455 mimics combined with wild type or mutant ULK2 transfection. (d) Effect of miR-4455 mimics combined with ULK2 overexpression plasmid transfection on the ULK2 expression in human podocytes revealed by western blotting. (e) Effect of miR-4455 mimics combined with ULK2 overexpression plasmid transfection on human podocytes activity by CCK8. (f) Effect of miR-4455 mimics combined with ULK2 overexpression plasmid transfection on apoptosis of human podocytes by flow cytometry (^∗∗^*p* < 0.01, ns means *p* > 0.05).

To identify targets of exosomal miR-4455, two bioinformatics tools (miRDB and TargetScan) were used to predict a set of common target genes. Among these, we verified that Unc-51 like autophagy activating kinase 2 (ULK2) is a direct target of miR-4455. First, sequence alignment between miR-4455 and full-length ULK2 showed that the 3′UTR of ULK2 may be a potential target of miR-4455 (Figure 4(a)). We found that the ULK2 protein expression was downregulated by miR-4455 in podocytes (Figure 4(b)). Wild-type and mutated miR-4455 binding sites were cloned into the luciferase vectors. We observed a marked decrease in luciferase activity in HEK293T cells cotransfected with the wild-type binding-site vector in the presence of miR-4455. However, cells containing the mutated binding-site vector did not exhibit such repression (Figure 4(c)). These results revealed that ULK2 was a direct target of miR-4455 in podocytes.

To determine the function of ULK2 in regulating podocyte injury, we upregulated the ULK2 expression by ULK2 overexpression plasmid transfection, and the effect was assessed by qRT-PCR and immunoblotting analyses (Figures 4(d) and 4(e)). We found that the ULK2 overexpression significantly increased podocyte activity and reduced apoptosis in podocytes, which was neutralized by miR-4455 mimics (Figures 4(e) and 4(f)). Taken together, these findings suggest that ULK2 is a direct downstream target of miR-4455 that mediates podocyte injury.

**Figure 5:**
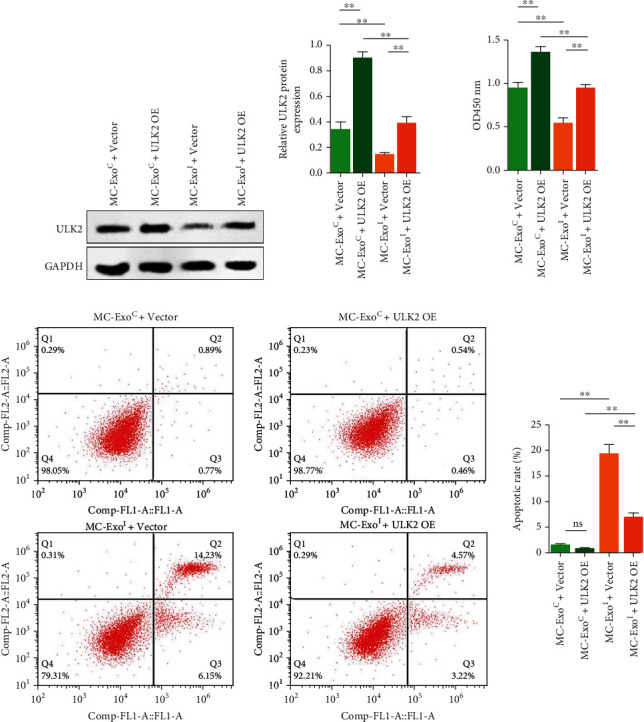
Effect of mesangial cell exosomes combined with ULK2 overexpression plasmid transfection on podocytes activity and apoptosis. (a) Effect of MC-ExoC or MC-ExoI combined with ULK2 overexpression plasmid transfection on the ULK2 expression in human podocytes revealed by western blotting. (b) Effect of MC-ExoC or MC-ExoI combined with ULK2 overexpression plasmid transfection on human podocytes activity by CCK8. (c) Effect of MC-ExoC or MC-ExoI combined with ULK2 overexpression plasmid transfection on apoptosis of human podocytes by flow cytometry (^∗∗^*p* < 0.01, ns means *p* > 0.05).

Subsequently, we used the ULK2 overexpression plasmid combined with MC exosomes to treat podocytes and evaluated their effects by qRT-PCR and immunoblotting analyses (Figures 5(a) and 5(b)). We found that the overexpression of ULK2 significantly inhibited the inhibitory effect of MC-ExoI on ULK2 expression in podocytes. In addition, the ULK2 overexpression increased podocyte activity and decreased podocyte apoptosis, which was neutralized by MC-ExoI mimics (Figures 5(b) and 5(c)). In summary, MC-ExoI induced podocyte injury by reducing the ULK2 expression in podocytes.

### 3.5. miR-4455 Inhibits Autophagy and Induces Podocyte Injury by Downregulating ULK2 Expression

**Figure 6:**
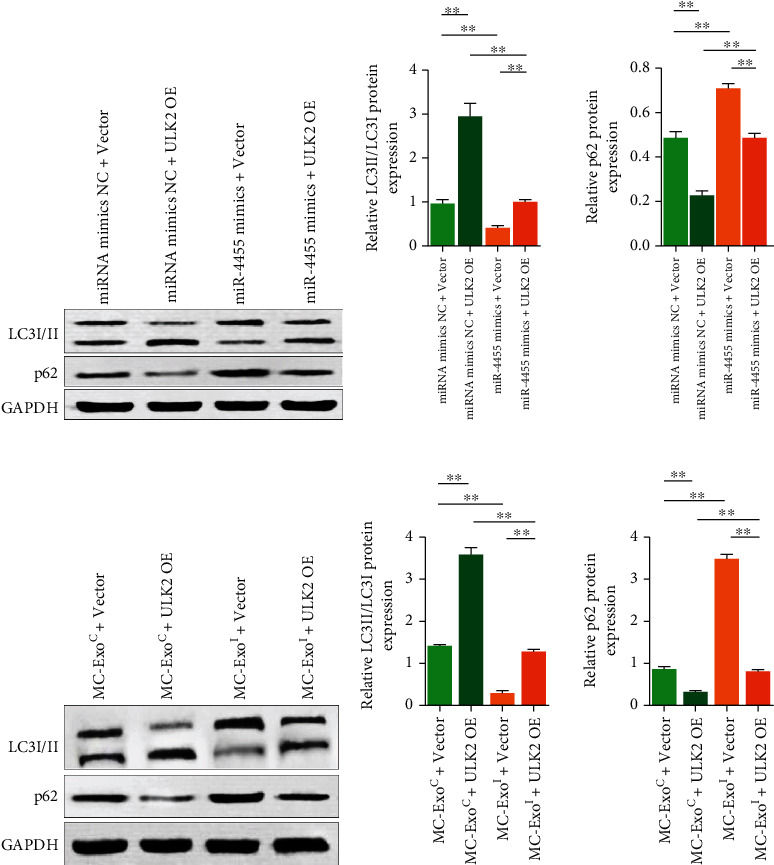
Effect of mesangial cell exosomes or miR-4455 mimics combined with ULK2 overexpression plasmid transfection on autophagy in podocytes. (a) Effect of miR-4455 mimics combined with ULK2 overexpression plasmid transfection on LC3II/I ratio and p62 protein content in human podocytes revealed by western blotting. (b) Quantitative plot of (a). (c) Effect of MC-ExoC or MC-ExoI combined with ULK2 overexpression plasmid transfection on LC3II/I ratio and p62 protein content in human podocytes revealed by western blotting. (d) Quantitative plot of (c) (^∗∗^*p* < 0.01, ns means *p* > 0.05).

ULK1/2 is a conserved serine/threonine phosphokinase that regulates autophagy by phosphorylating a series of substrates [20, 21]. Autophagy plays an important role in regulating podocyte function [22]. To analyze the effect of miR-4455 on the autophagy level of podocytes and determine whether it is related to changes in the ULK2 expression, we used miR-4455 mimics combined with ULK2 overexpression plasmid transfection to treat podocytes. Western blotting analysis showed that miR-4455 mimic treatment significantly reduced the proportion of LC3II/I in podocytes and increased p62 protein levels. ULK2 overexpression plasmid transfection significantly increased the LC3II/I ratio in podocytes treated with miRNA mimics NC or miR-4455 and reduced p62 protein content (*p* < 0.05, Figures 6(a) and 6(b)). This indicated that miR-4455 inhibits podocyte autophagy by downregulating ULK2.

Using MC exosomes combined with ULK2 overexpression plasmid–transfected podocytes, we found that MC-ExoI treatment significantly reduced the LC3II/I ratio and increased p62 protein content in podocytes. ULK2 overexpression plasmid transfection significantly increased the LC3II/I ratio and decreased p62 protein content in MC-ExoC or MC-ExoI-treated podocytes (*p* < 0.05, Figures 6(c) and 6(d)). These results indicate that MC-ExoI inhibited autophagy in podocytes through miR-4455 and depended on ULK2.

## 4. Discussion

IgA nephropathy, the most common primary glomerular disease worldwide, is a major cause of renal failure [1], and it has a complex and incompletely understood pathogenesis. Understanding the pathogenesis of IgA nephropathy can contribute to early IgAN diagnosis, providing the possibility of early treatment and stopping its progression to ESRD.

Since IgAN was initially reported, our understanding of its long-term prognosis, clinical and histological features, pathogenesis of onset and progression, risk factors for progression, and appropriate treatment under different clinical and histological conditions has steadily increased [23, 24]. The reduction in podocytes in IgAN has been reported to be closely related to the severity of glomerular disease [25], and autophagy is known to be important for podocyte maintenance [26]. Autophagy, the degradation of cytoplasmic content by lysosomal fusion, is precisely regulated and plays a crucial role in maintaining intracellular homeostasis [27], which is an evolutionarily conserved process that promotes internal environmental stability and intracellular defense [28]. ULK2, as the upstream autophagy induction factor, plays an essential role in autophagy regulation in mammalian cells [29]. Some studies have found that ULK2 can regulate autophagy by phosphorylating a series of substrates, such as VPS34 and beclin1 [20, 21].

Exosomes are rich in miRNAs that can be transmitted to target cells to regulate their function. The miR-4455 expression in gastric cancer tissues is downregulated, and miR-4455 can inhibit the growth and metastasis of gastric cancer cells [30, 31]. miRNAs, which are regulators of autophagy, have also been reported to regulate autophagy, for example, the effect of miRNA and autophagy on colorectal cancer [32]; miRNA-192-5p attenuates airway remodeling and autophagy in asthma by targeting MMP-16 and ATG7 [33]. The effects of miRNAs on the ULK2 expression have also been intensively studied in recent years; for example, miR-26a inhibits autophagy in porcine Sertoli cells by targeting ULK2 [34] and downregulated miRNA-26b inhibits laryngeal cancer proliferation by targeting ULK2 and inactivates the PTEN/AKT pathway [35]. Moreover, miR-26b inhibits prostate cancer by targeting ULK2 cell autophagy [36].

In our study, MCs treated with aIgA1 exhibited no change in exosome secretion, and the miR-4455 content in MC-ExoI was significantly increased. Subsequently, miR-4455 entered podocytes with exosome uptake, resulting in an increase in miR-4455 content in podocytes. miR-4455 reduced the ULK2 expression in podocytes by targeting ULK2, resulting in a decrease in the proportion of autophagy-related protein LC3II/I, an increase in autophagy-related protein p62, inhibition of autophagy in podocytes, and induction of podocyte injury.

## 5. Conclusions

The miR-4455 content in MC-ExoI was upregulated and increased with the uptake of exosomes into podocytes, which led to miR-4455 upregulation. miR-4455 reduced podocyte autophagy by targeting the ULK2 expression and ultimately induced podocyte injury. This suggests that miR-4455 is a regulatory miRNA for the ULK2 expression, affecting the autophagy level of podocytes through the miR-4455-ULK2 regulatory axis. Our findings elucidate a new target for IgAN treatment.

## Figures and Tables

**Table 1:** Primer sequences for real-time PCR.

Gene	Primer (5′-3′)
miR-6076	TGCGCAGCATGACAGAGGAGA
miR-1224-5p	TGCGCGTGAGGACTCGGGA
miR-146a-5p	TGCGCTGAGAACTGAATTCCAT
miR-4695-5p	TGCGCCAGGAGGCAGTGGGCGA
miR-214-5p	TGCGCTGCCTGTCTACACTTGC
miR-4455	TAATACAGGGTGTGTGT
miR universal primer	GTGCAGGGTCCGAGGT
U6 (forward)	CTCGCTTCGGCAGCACA
U6 (reverse)	AACGCTTCACGAATTTGCGT

**Table 2:** The growth/apoptosis reports of top 15 miRNAs.

miRNAs	Growth/apoptosis reports	References
miR-4499	Few related reports	
miR-6076	Inhibiting growth of endometrial carcinoma cells	doi:10.1002/jcb.29486
miR-1246	Promoting metastasis of colorectal cancer	doi:10.1186/s13046-019-1408-4
miR-1224-5p	Inhibiting growth of esophageal cancer cells	doi:10.1038/s41419-020-02801-6
miR-146a-5p	Inhibiting growth of pancreatic cancer cells	doi:10.7150/thno.40566
miR-4695-5p	Inhibiting growth of osteosarcoma cells	doi:10.3892/mmr.2018.9131
miR-4758-5p	Few related reports	
miR-3937	Few related reports	
miR-1306-3p	Few related reports	
miR-362-5p	Promoting growth of bladder cancer cells	doi:10.3389/fphar.2020.00164
miR-214-5p	Inhibiting growth of prostate cancer cells	doi:10.3233/CBM-190128
miR-572	Promoting growth of ovarian cancer cells	doi:10.1016/j.biopha.2015.12.005
miR-4455	Inhibiting growth of gastric cancer cells	doi:10.1186/s12935-018-0573-4
miR-6510-5p	Few related reports	
miR-4734	Few related reports	

## Data Availability

The authors confirm that the data supporting the findings of this study are available within the article.
